# Incidence and Prognostic Impact of *DNMT3A* Mutations in Korean Normal Karyotype Acute Myeloid Leukemia Patients

**DOI:** 10.1155/2015/723682

**Published:** 2015-01-11

**Authors:** Sang Hyuk Park, Jae-Cheol Choi, Shine Young Kim, Jongyoun Yi, Seung Hwan Oh, In-Suk Kim, Hyung-Hoi Kim, Chulhun Ludgerus Chang, Eun Yup Lee, Moo-Kon Song, Ho-Jin Shin, Joo Seop Chung

**Affiliations:** ^1^Department of Laboratory Medicine, Pusan National University School of Medicine, Pusan National University Hospital, 179 Gudeok-ro, Seo-gu, Busan 602-739, Republic of Korea; ^2^Biomedical Research Institute, Pusan National University Hospital, 179 Gudeok-ro, Seo-gu, Busan 602-739, Republic of Korea; ^3^Department of Laboratory Medicine, Hanmaeum Hospital, 21 Woni-daero, 682 Beon-gil, Seongsan-gu, Changwon-si, Gyeongsangnam-do 642-832, Republic of Korea; ^4^Department of Laboratory Medicine, Inje University College of Medicine, 75 Bokji-ro, Busanjin-gu, Busan 614-735, Republic of Korea; ^5^Department of Laboratory Medicine, Pusan National University School of Medicine, Pusan National University Yangsan Hospital, 20 Geumo-ro, Mulgeum-eup, Yangsan-si, Gyeongsangnam-do 626-770, Republic of Korea; ^6^Division of Hematology-Oncology, Department of Internal Medicine, Pusan National University School of Medicine, Pusan National University Hospital, 179 Gudeok-ro, Seo-gu, Busan 602-739, Republic of Korea

## Abstract

*Background*. *DNA methyltransferase 3A* (*DNMT3A*) mutation was recently introduced as a prognostic indicator in normal karyotype (NK) AML and we evaluated the incidence and prognostic impact of *DNMT3A* mutations in Korean NK AML patients. *Methods*. Total 67 NK AML patients diagnosed during the recent 10 years were enrolled. *DNMT3A* mutations were analyzed by direct sequencing and categorized into nonsynonymous variations (NSV), deleterious mutations (DM), and R882 mutation based on *in silico* analysis results. Clinical features and prognosis were compared with respect to *DNMT3A* mutation status. *Results*. Three novel (I158M, K219V, and E177V) and two known (R736H and R882H) NSVs were identified and the latter three were predicted as DMs. *DNMT3A* NSVs, DMs, and R882 mutation were identified in 14.9%–17.9%, 10.3%–10.4%, and 7.5% of patients, respectively. *DNMT3A* mutations were frequently detected in *FLT3* ITD mutated patients (*P* = 0.054, 0.071, and 0.071 in NSV, DMs, and R882 mutation, resp.) but did not affect clinical features and prognosis significantly. *Conclusions*. Incidences of *DNMT3A* NSVs, DMs, and R882 mutation are 14.9%–17.9%, 10.3%–10.4%, and 7.5%, respectively, in Korean NK AML patients. *DNMT3A* mutations are associated with *FLT3* ITD mutations but do not affect clinical outcome significantly in Korean NK AML patients.

## 1. Introduction

Acute myeloid leukemia (AML) is a clonal hematologic disorder exhibiting heterogeneous prognosis based on the results of cytogenetic and molecular aberration analysis [1, 2]. Among patients with AML, approximately half of them are categorized into the intermediate prognosis group defined in the recent National Comprehensive Cancer Network (NCCN) guidelines [3]. In AML patients with intermediate prognosis group, most of them show normal karyotype (NK) and the prognosis of these patients generally depends on molecular aberrations and fms-related tyrosine kinase 3 gene internal tandem duplications (*FLT3* ITD) which are detected in 30% of AML patients and possess poor prognosis regardless of mutation status in other genes [4–8], nucleophosmin (*NPM1*), and CCAAT/enhancer binding protein *α* (*CEBPA*) mutations which are detected in 50% and 13–15% of NK AML, respectively, and exhibit good prognosis [9–15] having been well established based on various previous studies. In addition, the mutations in isocitrate dehydrogenase (*IDH*)1 and 2 genes, which are identified in 6.0%–15.9% of AML patients, were recently introduced as an unfavorable prognostic indicator in* NPM1 *mutated AML patients [16–19].

Besides these mutations described above, recent studies based on the whole-genome sequencing approach found a new prognostic marker of mutations in* DNA methyltransferase 3A* (*DNMT3A*) gene involving DNA methylation, which is the most important epigenetic process in the regulation of gene expression and chromatin structural remodeling, in the patients with NK AML [20–23].* DNMT3A* mutations have been reported in 18–22% of AML (29–34% of CN-AML) [24–27] and previous studies showed that the mutations of* DNMT3A* in AML are frequently found in the patients with* NPM1 *mutations and have the mutation hotspot of arginine in 882th codon (R882) [20–27]. Some previous studies reported that the patients with* DNMT3A* mutations show inferior overall survival (OS) compared to those without mutations [20, 21, 25] and another recent study reported a trend of more frequent relapse and inferior OS in the patients who achieved complete remission (CR) when they possess* DNMT3A* mutations [27]. However, in contrast to* NPM1* and* CEBPA* mutations in which the prognostic value was confirmed and which were included as a provisional entity in the 2008 WHO classifications, the prognostic value of* DNMT3A *mutations as well as* IDH1/2* mutations has not been confirmed up to present time. In addition, although two recent studies reported poor prognostic impact of* DNMT3A* mutations in Chinese and Taiwanese AML patients [28, 29], studies which analyzed the clinical relevance of* DNMT3A *mutations in Asian population are outnumbered compared to Western population and there have been no reports on the clinical impact of* DNMT3A* mutations in Korean AML patients.

In this study, we performed direct sequencing of all 23 exons in* DNMT3A* gene in 39 NK AML patients who were diagnosed at single tertiary hospital in Korea and analyzed the mutation characteristics. In addition, we performed* in silico* analysis to predict the deleterious effect of detected nonsynonymous variations on the protein function and structure and estimated the clinical relevance of* DNMT3A* mutations on the prediction of clinical course while including another 28 NK AML patients.

## 2. Materials and Methods

### 2.1. Patients Selection

A total of 60 NK AML patients who were diagnosed from January 2004 to July 2010 and treated in Pusan National University Hospital were initially recruited from the retrospective review of electronic medical record (EMR), and a total of 39 patients in whom the quality of extracted bone marrow (BM) DNA at diagnosis was sufficient to be analyzed by direct sequencing were finally enrolled in the first study cohort. In these 39 patients, direct sequencing of all 23 exons in* DNMT3A* gene was performed to analyze the incidence and distribution characteristics of all detected variations in* DNMT3A* gene. The median age and follow-up interval of first study cohort was 39.0 years (range: 11.0 years–83.0 years) and 5.2 months (range: 0.0 months–72.0 months), respectively, and this cohort included 25 male (64.1%) and 14 female (35.9%) patients.

In addition, total 28 NK AML patients who were diagnosed from August 2010 to March 2014 at the same hospital were additionally enrolled as second study cohort to evaluate the clinical and prognostic impact of mutation hotspot R882 in* DNMT3A* gene. In these patients, direct sequencing of* DNMT3A* exon 23 was performed. The median age and follow-up interval of second study cohort were 28.0 years (range: 15.0 years–80.0 years) and 6.4 months (range: 0.1 months–42.6 months), respectively, and this cohort included 12 male (42.9%) and 16 female (57.1%) patients.

### 2.2. Patients Treatment

All these patients received induction chemotherapy with cytarabine 100 mg/m^2^/day for seven days plus daunorubicin 45 mg/m^2^/day for three days (the AD regimen) or cytarabine 100 mg/m^2^/day for seven days plus idarubicin 12 mg/m^2^/day for three days (the AI regimen). CR was defined by less than 5% of residual leukemic blasts in the BM aspirates and more than 20% of BM cellularity, normal maturation of all cellular components (erythrocytic, granulocytic, and megakaryocytic series) in the BM aspirates, normal values for absolute neutrophil counts (>1,000/*μ*L) and platelet counts (>100,000/*μ*L) after the first or second course of induction chemotherapy. Relapse was defined as the presence of ≥5% leukemic blasts in BM aspirates for patients who had previously achieved CR. Stem cell transplantation (SCT) after first CR achievement were considered as a final treatment modality depending on the patient's age, availability of a suitable donor, and* FLT3* ITD mutation status.

As a prognosis indicator, CR, relapse, and death rates were used and both OS and event-free survival (EFS) were additionally calculated. OS was defined as the interval from diagnosis to death or last follow-up. EFS was defined as the interval from CR to relapse or death for patients who were relapsed or expired during follow-up or the interval from CR to last follow-up for surviving patients who were not relapsed during follow-up interval. If the patient underwent SCT, the CR date was replaced by the SCT date in the calculation of EFS. This study was approved by the institutional review board of author's institution.

### 2.3. Mutation Analysis of* DNMT3A* Gene

DNA samples were extracted from the unstained remnant smear slides of BM aspirates or chromosomal cell pellets obtained at diagnosis using DNA Blood Mini extraction kit (Qiagen, Hilden, Germany), as recommended by manufacturer. All 23 exons of* DNMT3A* gene were amplified with primers listed in Table 1. All forward and reverse primers had M13F binding tag (tgtaaaacgacggccagt) and M13R binding tag (caggaaacagctatgacc) on the 5′ ends of each primer. Polymerase chain reactions (PCR) were conducted with hot start procedure according to the manufacturer's instructions (i-StarTaq, INTRON Biotechnology, Sungnam, Korea) described below: an initial denaturation at 95°C for 5 minutes and by 30 cycles of following denaturation at 95°C for 30 seconds, annealing at 60°C for 30 seconds, and extension at 72°C for 30 seconds. A final extension was done at 72°C at 5 minutes on Verity-96 well thermal cycler PCR system (Applied Biosystems Inc., Foster City, CA, USA).

PCR products were purified with AccuPrep kit (Bioneer, Daejeon, Korea) and were used in the sequencing reaction using BigDye Terminator v.3.1 Cycle Sequencing Ready Reaction kit (Applied Biosystems Inc., USA). Direct sequencing of all 23 exons was performed with ABI 3130 Genetic Analyzer (Applied Biosystems Inc., USA). The SeqScape software version 2.6 (Applied Biosystems Inc., USA) was used to align the obtained and reference sequences (NM_175629.2) and all detected nonsynonymous and synonymous variations were described according to the guidelines of the Human Gene Variation Society (HGVS; http://www.hgvs.org/).

### 2.4. *In Silico* Analysis to Predict the Deleterious Effect of All Detected Nonsynonymous Variations on the Protein Function and Structure

To predict the deleterious effect of novel and known sequence variations, all detected nonsynonymous variations were submitted to three web-based programs: Sorting Intolerant From Tolerent (SIFT, http://sift.jcvi.org/), Polyphen-2 (version 2.2.2, http://genetics.bwh.harvard.edu/pph/), and MutationTaster (http://www.mutationtaster.org/). The web-based database dbSNP (http://www.ncbi.nlm.nih.gov/projects/SNP/) was applied in the confirmation of known SNP and the novelty of detected nonsynonymous variations was validated by web-based database Catalogue of Somatic Mutations in Cancer (COSMIC, http://cancer.sanger.ac.uk/cancergenome/projects/cosmic/). Cn3D macromolecular structure viewer was used to annotate the mutation sites in 3D structure [30].

### 2.5. Comparison of Clinical Characteristics and Prognosis in Patients with respect to Mutation Status of* DNMT3A *Gene

To assess the clinical relevance of* DNMT3A* mutation analysis, the comparison of clinical characteristics and prognosis in NK AML patients with respect to mutation status of* DNMT3A* genes was performed. All patients were categorized into two subgroups with respect to nonsynonymous variation status of* DNMT3A* gene and both clinical features and prognosis were compared between two subgroups to evaluate the clinical relevance of nonsynonymous variations of* DNMT3A* gene. Subsequently, nonsynonymous variations which were predicted as deleterious mutation in at least two of three* in silico* analyses were recruited and identical comparisons were performed again between patients with deleterious mutations and those without deleterious mutations to further evaluate the clinical relevance of deleterious mutations in* DNMT3A* gene.

Finally, to evaluate the clinical relevance of* DNMT3A* R882 mutation hotspot, all patients were categorized into two subgroups with respect to* DNMT3A* R882 mutation status, and identical comparisons were performed again. Clinical data of patients were obtained from retrospective review of EMR and included age and gender, complete blood cell (CBC) counts, blasts (%) in BM aspirates,* FLT3* ITD and D835 mutation status (at diagnosis), and date of CR, relapse, and death (during follow-up).

### 2.6. Multivariate Analysis of Overall Survival

For the identification of prognostic impact in* DNMT3A *mutations in association with other possible prognostic indicators such as* FLT3* ITD mutation status, age, BM blast (%), and SCT performance status, the multivariate analysis was performed when the clinical variables mentioned above were included as covariables.

### 2.7. Statistical Analysis

Pearson chi-square yest or Fisher's exact test (for small numbers less than 5) were applied to compare categorical variables (gender, CR rates, relapse rates, and death rates) between each two patient subgroups. Mann-Whitney *U* test was used to compare continuous variables (age, CBC counts at diagnosis and interval from diagnosis to CR) between each two patient subgroups. Estimates of OS and EFS were generated using Kaplan-Meier methods and were compared using a log-rank test. Multivariate analyses of OS were performed with a Cox's proportional hazards model. For all analyses, tests were two-tailed and *P* values ≤ 0.05 and 0.05–0.10 were considered statistically significant and marginally significant, respectively. All calculations were performed using SPSS 13.0.1 for Windows (SPSS Inc, Chicago, IL, USA).

## 3. Results

### 3.1. Summary of Detected Variations in* DNMT3A* Gene

Among 39 patients included in the first study cohort, two known synonymous variations (c.27C>T (rs41284843, minor allele frequency = 0.0851) and c.1266G>A (rs2276598, minor allele frequency = 0.2337)) and one novel synonymous variation (c.2043C>G) were detected in 5, 19 (including 2 homozygotes), and 6 patients (12.8%, 48.7%, and 15.4%), respectively.

**Figure 1:**
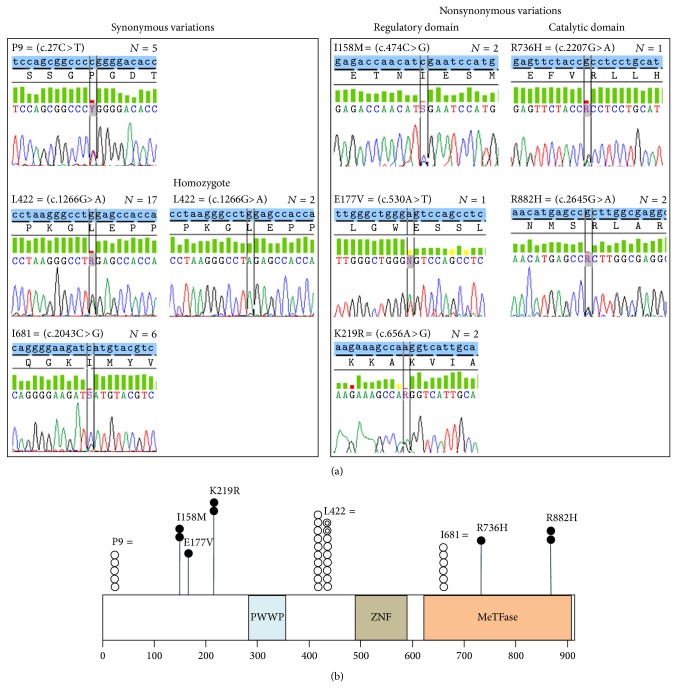
Electropherograms of all* DNMT3A *variations including synonymous and nonsynonymous variations observed in this study (a) and schematic summary representing locations and frequency of all detected* DNMT3A *variations (b). Note: (a) shows electropherogram of all detected* DNMT3A* variations in 39 patients included in the first study cohort. (b) shows schematic summary of all detected* DNMT3A *variation including location and frequency. The number of circles indicates the frequency of detected variations. Closed black and white circle indicates heterozygous nonsynonymous and synonymous variation case, respectively, and closed double-layered white circle indicates homozygous synonymous variation case. Abbreviations: P, proline; I, isoleucine; M, methionine; R, arginine; H, histidine; L, leucine; E, glutamate; V, valine; K, lysine; PWWP, Pro-Trp-Trp-Pro motif; ZNF, zinc finger; MeTFase, methyltransferase.

Total five different nonsynonymous variations, which are all missense mutations, were detected in seven (17.9%) patients and included two known mutations (c.2207G>A (p.Arg736His, COSM133737, detected in one patient) and c.2645G>A (p.Arg882His, COSM52946, detected in two patients)) and three novel mutations (c.474C>G (p.Ile158Met, detected in two patients), c.530A>T (p.Glu177Val, detected in one patient), and c.656A>G (p.Lys219Arg, detected in two patients)). All 7 patients with* DNMT3A* mutations harbored single mutation, except for one patient who showed both K219R and R736H mutations simultaneously. All 2 known mutations (R736H and R882H) induced protein change in the catalytic domain of DNMT3A protein which has critical role in methyltransferase activity, and all three novel mutations (I158M, E177V, and K219R) were involved in protein change at the regulatory domain of DNMT3A protein. These results are summarized in Figure 1.

Subsequently performed direct sequencing of* DNMT3A* exon 23 in 28 NK AML patients who were included in second study cohort detected additional three R882H mutations. When summarizing these results, the incidence of* DNMT3A *nonsynonymous variations was calculated to be 7/39 (17.9%) in the first study cohort and finally estimated to be 10/67 (14.9%) when adding data from 28 patients in the second study cohort, and the frequency of* DNMT3A *R882 mutations was exactly calculated to be 5/67 (7.5%).

### 3.2. *In Silico* Analysis Results to Predict the Deleterious Effect of Detected Nonsynonymous Variations on the Protein Function and Structure

Among five* DNMT3A* nonsynonymous variations detected in this study, R882H was predicted as a deleterious mutation by all three web-based prediction programs, as reported in the previous study [21] and another known variation (R736H) was also predicted as a deleterious mutation by two programs. In addition, one novel variation (E177V) was also predicted as a deleterious mutation by all three programs. In contrast, two novel variations (I158M and K219R) were predicted as a benign or tolerated variation in all three programs. These results are represented in Table 2. With these results, the frequency of deleterious mutation of* DNMT3A *gene was calculated to be 4/39 (10.3%) in the first study cohort and finally estimated to be 7/67 (10.4%) when adding data from 28 patients in the second study cohort.

In the 3D structure model of methyltransferase domains, R882 residue was located in the region interfacing with two DNMT3A molecules, which is consistent with the results of previous literature [21], and R736 residue was located on the opposite side and toward DNMT3L molecule. These results are illustrated in the Supplemental Figure 1  available online at http://dx.doi.org/10.1155/2015/723682.

### 3.3. Comparison of Clinical Characteristics and Prognosis in Patients with respect to Mutation Status of* DNMT3A *Gene

**Figure 2:**
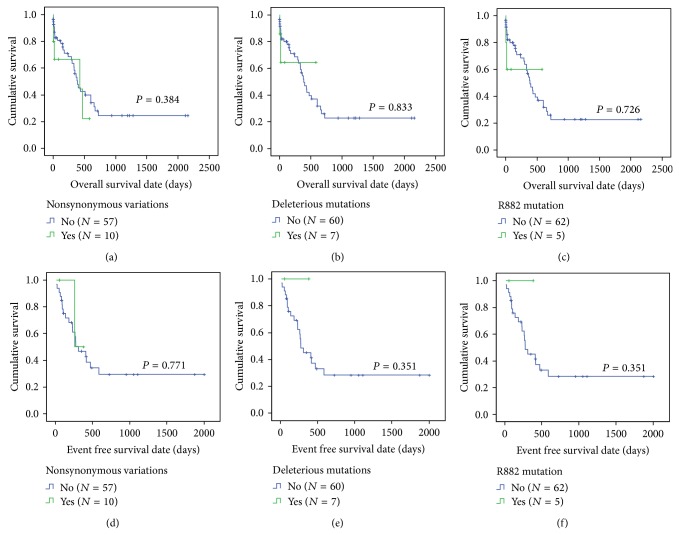
Comparisons of overall survival and event-free survival in total 67 NK AML patients with respect to* DNMT3A* nonsynonymous variations ((a) and (d)), deleterious mutations ((b) and (e)), and R882 mutation ((c) and (f)), respectively. Abbreviation: R, arginine.

In the comparison of patients with* DNMT3A* nonsynonymous variations and those without nonsynonymous variations, both clinical features and prognosis (OS and EFS) were not significantly different between two patient subgroups, except for the trend of high* FLT3* ITD mutation frequency in patients with* DNMT3A* nonsynonymous variations than those who do not harbor (66.7% versus 24.4%, *P* = 0.054). Identical results were found in the comparison with respect to* DNMT3A* deleterious mutation status, since the patients with* DNMT3A* deleterious mutations also showed the trend of more frequent* FLT3* ITD mutation than those without* DNMT3A* deleterious mutations (75.0% versus 25.5%, *P* = 0.071). Additionally performed comparison between patients with* DNMT3A* R882 mutation and those without R882 mutation also showed identical results again. These results are summarized in Table 3 and Figure 2.

### 3.4. Multivariate Analysis of Overall Survival

The multivariate analysis of OS in total 67 NK AML patients demonstrated that the presence of* FLT3* ITD mutation is a significantly poor prognostic indicator of OS (hazard ratio 2.798, *P* = 0.041) and the performance of SCT during follow-up is a significantly good prognostic indicator of OS (hazard ratio 0.118, *P* = 0.001) as expected. However, the presence of* DNMT3A* nonsynonymous variations (hazard ratio 1.229, *P* = 0.724), deleterious mutations, and R882 mutations (hazard ratio 2.285, *P* = 0.313 in both) did not affect OS significantly as well as increasing age and BM blasts (%), although the trend was toward to adverse prognosis. These results are described in Table 4.

## 4. Discussion

Previous studies that analyzed* DNMT3A* mutations showed that the incidence of* DNMT3A* mutation is 18–22% of AML and 29–34% of NK AML [24–27], including the largest study which analyzed 489 AML patients and reported the frequency of* DNMT3A* mutation as 17.8% of AML patients and 27.2% of NK AML patients [24]. In Korean population, although the data of clinical outcome was not provided, a recent study reported the incidence of* DNMT3A* R882 mutation as 9.4% in adulthood AML [31]. Our present study showed that the incidence of* DNMT3A* nonsynonymous variations is 17.9% in first study cohort and 14.9% in extended cohort, which is similar to the results from previous studies [24–27]. In addition, our present study analyzed the incidence of* DNMT3A* R882 mutation as 7.5%, which is also similar to the previous data in Korean population [31]. All these results demonstrate that* DNMT3A* mutation can be also considered as a frequently occurring nonrandom mutation in Korean NK AML patients.

Our present study applied direct sequencing of all 23 exons in* DNMT3A* gene to identify all possible variations affecting* DNMT3A* protein function and structure and could detect total 8 different sequence variations, including 3 synonymous and 5 nonsynonymous variations. Among three synonymous variations, two known variations (c.27C>T and c.1266G>A) of which minor allele frequencies are 8.51% and 23.37% were found with the frequency of 12.8% and 48.7%, respectively, and therefore these two variations can be considered as constitutional single nucleotide polymorphisms (SNP). In addition, our present study could detect one novel synonymous variation (c.2043C>G), with the incidence of 15.4% and this novel SNP can be listed in the current SNP databases.

For the nonsynonymous variations, we could find 3 different novel variations (I158M, E177V, and K219V) in the regulatory domains of DNMT3A protein that have a role in interaction with other molecules and protein localization to the nuclei, as well as 2 known variations (R736H and R882) located in the catalytic methyltransferase domain of DNMT3A protein. In addition,* in silico* analysis performed in our study predicted not only both R736H and R882H variations in catalytic domain but also a novel E177V variation in regulatory domain to be deleterious to protein function, and previously known mutation hotspot R882 was not frequently detected in our present study. These results can be novel findings of our present study since R882 residue of* DNMT3A* gene has been regarded as a frequently mutated hotspot in previous studies [20, 21, 24–27], which would have a gain-of-function activity or dominant negative effects [20]. All these results may suggest the presence of ethnic variation in the characteristics of* DNMT3A* mutations and justify the application of detection method which covers all exons of* DNMT3A* gene rather than mutation hotspot R882 in Korean population.

Our structural analysis using 3D model showed that R736 residue is exposed to the DNMT3L molecules that are a stimulating factor of DNMT3A and a mediator of transcriptional repression through interaction with histone deacetylase 1 [30]. Therefore, it may be considered that the alteration of R736 residue may affect the efficiency and regulation of DNMT3A protein function, as similarly with previously demonstrated R882 residue [21]. In contrast, in the regulatory domain of DNMT3A protein, only E177V was identified as deleterious mutation and we could not provide the possible explanation for this result in our present study. More extensive functional analysis on the DNMT3A protein should be required for the clarification of this finding.

*DNMT3A* mutations have been reported to occur in patients with* NPM1* and* FLT3* mutations [24, 25, 27] but the clinical and prognostic impact of* DNMT3A* mutations has not been confirmed yet. Some studies revealed the poor prognostic effect of* DNMT3A* mutations on survival outcome, both in overall [25] and in high risk (*FLT3* ITD mutated but NPM1 nonmutated) NK AML patients [24]. Two studies performed in Asian population also reported poor prognostic impact of* DNMT3A* mutations in NK AML [28, 29]. In contrast, other studies also reported no significant prognostic effect of* DNMT3A* mutations in AML patients with intermediate risk group [27] and in low risk (*NPM1* mutated but* FLT3 *ITD unmutated) NK AML patients [24]. A recent study reported that the poor prognostic relevance of* DNMT3A *mutation is dependent on age and its mutation type (R882 versus non-R882) [26]. Our present study could also demonstrate a trend of association between* FLT3* ITD mutation and* DNMT3A* mutations in terms of mutation frequency, as reported in the previous literature [24, 25, 27]. However, our present study could not demonstrate a significant effect of* DNMT3A* mutations on both clinical features and prognosis, regardless of mutation type (nonsynonymous variations, deleterious mutations, or R882 mutation hotspot) in Korean NK AML patients although the trend was toward adverse prognosis. This result may support previously given hypothesis in our present study referring the presence of ethnic variation in the characteristics of* DNMT3A* mutations. In addition, from our present study result it can be speculated that the adverse prognostic effect of* DNMT3A* mutations may not exist in overall Korean NK AML patients and although some variations are predicted to be deleterious to protein function in our study, the adverse prognostic impact may reside in all non-synonymous variations of* DNMT3A* gene and, therefore, all 23 exons of* DNMT3A *gene should be screened for the detection of clinically important* DNMT3A* mutations.

Our study has some important limitations in terms of small patient numbers in each subgroup and mutation analysis of other genes with clinical significance. First, our study cohort included total 67 patients (39 in first and 28 in second study cohort) who were diagnosed and treated in single tertiary hospital, and statistical power in the comparison of clinical features and prognosis with respect to* DNMT3A* mutation status, especially in the analysis focused on* FLT3* ITD mutated patients, is estimated to be significantly low. Therefore, our present results should be interpreted with caution and regarded to be preliminary data providing future issues to be addressed. Second, although both* NPM1 *and* CEBPA* mutations have prognostic value in NK AML and some previous study also suggested the association of* NPM1* and* DNMT3A* mutations [24, 25, 27], our present study did not include the data of* NPM1* and* CEBPA *mutation status and, therefore, comprehensive analysis in terms of mutational relations among three genes (*NPM1*,* CEBPA*, and* DNMT3A*) could not be performed. More large scaled and comprehensive study with these data should be required in the future to address these points.

## 5. Conclusions

Our present study identified three novel (I158M, E177V, and K219V in the regulatory domain) and two known (R736H and R882H in the methyltransferase domain)* DNMT3A* nonsynonymous variations, and three (E177V, R736H and R882H) of them were predicted to be deleterious to protein function and structure. The incidences of* DNMT3A* nonsynonymous variations, deleterious mutations, and R882 mutation were 14.9%–17.9%, 10.3%–10.4%, and 7.5%, respectively, in Korean NK AML patients, without predominance of known mutation hotspot R882.* DNMT3A *mutations were frequently detected in* FLT3 *ITD mutated NK AML patients but did not significantly affect clinical features and prognosis in Korean NK AML patients, and this issue needs to be further evaluated in the future.

## Supplementary Material

Schematic 3D structure of DNMT3A protein and detailed site annotation of R736 and R882 residue

## Figures and Tables

**Table 1:** The forward and reverse primer sequences used in the amplification of all 23 exons in *DNMT3A* gene.

Primer	Primer sequence
>DNMT02F	tgtaaaacgacggccagtTCCAAAGACCACGATAATTCCTTC
>DNMT03F	tgtaaaacgacggccagtCTGGAATGCTACACTGCTGGG
>DNMT04F	tgtaaaacgacggccagtACTTGGAGAAGCGGAGTGAGC
>DNMT05F	tgtaaaacgacggccagtGGATGTGTAAAGAAGGAGGAGGG
>DNMT06F	tgtaaaacgacggccagtACATTGTGTTTGAGGCGAGTGC
>DNMT07F	tgtaaaacgacggccagtCTAATTCCTGGAGAGGTCAAGGTG
>DNMT08F	tgtaaaacgacggccagtTCTTGCCTCATTCAGATGGAGC
>DNMT09F	tgtaaaacgacggccagtGTGCTTGCAAGTGTAAGCCTCG
>DNMT10F	tgtaaaacgacggccagtCTTGAGCCTGACCCATCTGC
>DNMT11F	tgtaaaacgacggccagtTTCCTGTCAGCCTGTAACTGACC
>DNMT12F	tgtaaaacgacggccagtTTATTGATGAGCGCACAAGAGG
>DNMT13F	tgtaaaacgacggccagtAGGGAGAGGCCCTTCGGTGG
>DNMT14F	tgtaaaacgacggccagtGGTCATGTCTTCAGGGCTTAGG
>DNMT15F	tgtaaaacgacggccagtTTTCCATTCCAGGTAGCACACC
>DNMT16F	tgtaaaacgacggccagtAGGGTGTGTGGGTCTAGGAGC
>DNMT17F	tgtaaaacgacggccagtGACTTGGGCCTACAGCTGACC
>DNMT18F	tgtaaaacgacggccagtATAGGACAGTGGTGTGGCTCG
>DNMT19F	tgtaaaacgacggccagtGACAGCTATTCCCGATGACCC
>DNMT20F	tgtaaaacgacggccagtGCCGGCGCTGTTTCATGC
>DNMT21F	tgtaaaacgacggccagtCCTTCCCGCTGTTATCCAGG
>DNMT22F	tgtaaaacgacggccagtTGGCATATTTGGTAGACGCATGAC
>DNMT23F	tgtaaaacgacggccagtTCCCAGTCCACTATACTGACGTCTC
>DNMT91F	tgtaaaacgacggccagtACAAAGAAAATGTTCCCTCCCTCC
>DNMT92F	tgtaaaacgacggccagtAAATCTGGTATGGTGGAAATGGG
>DNMT02R	caggaaacagctatgaccTCCCTCTCCCAGGCCAGA
>DNMT03R	caggaaacagctatgaccATACATCACTGCCATCGACAGG
>DNMT04R	caggaaacagctatgaccAAGCAGACCTTTAGCCACGACC
>DNMT05R	caggaaacagctatgaccGAACAGCTAAACGGCCAGAGG
>DNMT06R	caggaaacagctatgaccACTGAGGCCCATCACTTCTGG
>DNMT07R	caggaaacagctatgaccAGATGGAGAGAGGAGAGCAGGAC
>DNMT08R	caggaaacagctatgaccCCTGGGATCAAGAACCTTCCC
>DNMT09R	caggaaacagctatgaccCCTGCACTCCAACTTCCAGG
>DNMT10R	caggaaacagctatgaccGCAGGTCATTCAAGTCCTGACC
>DNMT11R	caggaaacagctatgaccATGCAGGCCTCCTGGTGC
>DNMT12R	caggaaacagctatgaccTCCCATGTCATTCAAACCTTCC
>DNMT13R	caggaaacagctatgaccACACAGTCAGCCAGAAGGCCG
>DNMT14R	caggaaacagctatgaccTGCTACCTGGAATGGAAAGACC
>DNMT15R	caggaaacagctatgaccAGGCTCCTAGACCCACACACC
>DNMT16R	caggaaacagctatgaccGCTGTGAAGCTAACCATCATTTCG
>DNMT17R	caggaaacagctatgaccAAATGAAAGGAGGCAAGGGC
>DNMT18R	caggaaacagctatgaccTTCTTCCTGTCTGCCTCTGTCC
>DNMT19R	caggaaacagctatgaccTGCAGATGAGACAGGATGAAGC
>DNMT20R	caggaaacagctatgaccCCACTATGGGTCATCCCACCTGC
>DNMT21R	caggaaacagctatgaccCATCCTGCCCTTCCTTCTCC
>DNMT22R	caggaaacagctatgaccTGGGAAATGCTTGATAAAACCCAC
>DNMT23R	caggaaacagctatgaccTCTCTCCATCCTCATGTTCTTGG
>DNMT91R	caggaaacagctatgaccCCAGCACTAAGTCAGCATCTCCAG
>DNMT92R	caggaaacagctatgaccTGGAGTTCTTATGGATCACACCC

DNMT3A: DNA methyltransferase 3A; F: forward; R: reverse.

**Table 2:** *In silico* analysis results of three web-based programs to predict the deleterious effect of detected nonsynonymous variations on the protein function and structure.

Mutations	Sequence variation database	SIFT		Polyphen-2		MutationTaster	
		Score	Prediction	Score	Prediction	Score	Prediction
I158M	Novel	0.21	Tolerated	0.191	Benign	0.27	Polymorphism
E177V	Novel	0.02	Damaging	0.981	Probably damaging	3.30	Disease causing
K219R	Novel	0.71	Tolerated	0.002	Benign	0.71	Polymorphism
R736H	rs139293773 COSM133737	0.39	Tolerated	0.997	Probably damaging	0.79	Disease causing
R882H	rs147001633 COSM52946	0.03	Damaging	0.651	Possibly damaging	0.79	Disease causing

I: isoleucine; M: methionine; E: glutamate; V: valine; K: lysine; R: arginine; H: histidine.

**Table 3:** Comparison of demographic and clinical features in all 67 NK AML patients with respect to *DNMT3A* nonsynonymous variation status.

Clinical characteristics	*DNMT3A* nonsynonymous variation status		*P* value	*DNMT3A *deleterious mutation status		*P* value	*DNMT3A *R882 mutation status		*P* value
	No variation (*N* = 57)	Variation (*N* = 10)		No mutation (*N* = 60)	Mutation (*N* = 7)		No mutation (*N* = 62)	Mutation (*N* = 5)	
Sex (M : F)^*^	33 : 24	4 : 6	NS	34 : 26	3 : 4	NS	35 : 27	2 : 3	NS
Age, years, median (range)^**^	56.0 (11.0–80.0)	62.0 (18.0–83.0)	NS	56.0 (11.0–80.0)	59.0 (18.0–83.0)	NS	56.0 (11.0–83.0)	59.0 (18.0–77.0)	NS
Laboratory findings at diagnosis, median (range)									
WBC (×10^9^/L)^**^	35.6 (0.9–368.9)	63.8 (0.6–282.2)	NS	36.3 (0.6–368.9)	55.5 (0.9–206.0)	NS	33.6 (0.6–368.9)	72.2 (0.9–206.0)	NS
Hb (g/dL)^**^	8.6 (5.4–14.2)	7.7 (5.6–10.7)	NS	8.6 (5.4–14.2)	7.3 (5.6–10.7)	NS	8.6 (5.4–14.2)	7.3 (5.6–10.7)	0.097
Platelets (×10^9^/L)^**^	54.0 (5.0–172.0)	62.0 (38.0–270.0)	NS	54.0 (5.0–172.0)	96.0 (43.0–270.0)	NS	53.5 (5.0–172.0)	113.0 (51.0–270.0)	NS
BM blasts (%)^**^	73.0 (22.1–97.0)	74.3 (29.5–96.2)	NS	72.9 (22.1–97.0)	75.0 (29.5–84.3)	NS	72.9 (22.1–97.0)	75.0 (29.5–84.3)	NS
*FLT3* ITD mutation (%)^*^	11/45 (24.4)	4/6 (66.7)	0.054	12/47 (25.5)	3/4 (75.0)	0.071	12/47 (25.5)	3/4 (75.0)	0.071
*FLT3* D835 mutation (%)^*^	2/45 (4.4)	0/6 (0.0)	NS	2/47 (4.3)	0/4 (0.0)	NS	2/47 (4.3)	0/4 (0.0)	NS
CR rates (%)^*^	33/57 (57.9)	3/10 (30.0)	NS	34/60 (56.7)	2/7 (28.6)	NS	34/62 (54.8)	2/5 (40.0)	NS
Interval from Dx to CR, days, median (range)^**^	34.0 (21.0–83.0)	53.0 (25.0–164.0)	NS	35.0 (21.0–164.0)	39.0 (25.0–53.0)	NS	35.0 (21.0–164.0)	39.0 (25.0–53.0)	NS
Relapse rates (%)^*^	16/33 (48.5)	1/3 (33.3)	NS	17/34 (50.0)	0/2 (0.0)	NS	17/34 (50.0)	0/2 (0.0)	NS
Death rates (%)^*^	31/57 (54.4)	5/10 (50.0)	NS	34/60 (56.7)	2/7 (28.6)	NS	34/62 (54.8)	2/5 (40.0)	NS
SCT performance rates (%)^*^	19/57 (33.3)	1/10 (10.0)	NS	19/60 (31.7)	1/7 (14.3)	NS	19/62 (30.6)	1/5 (20.0)	NS

Note: *P* values were obtained from Chi-square test/Fisher's exact test^*^ and Mann-Whitney *U* test^**^.

WBC: white blood cell; Hb: hemoglobin; BM: bone marrow; FLT3: fms-related tyrosine kinase 3; ITD: internal tandem duplications; D: aspartate; CR: complete remission; Dx: diagnosis; SCT: stem cell transplantation; DNMT3A: DNA methyltransferase 3A; R: arginine; NS: not significant.

**Table 4:** Multivariate analysis of overall survival.

Variables	Overall survival		
	HR (95% CI)	*P* value	Prognostic impact
All 67 patients			
*FLT3 *ITD mutation (compared with wild type)	2.798 (1.041–7.517)	0.041	Poor
*DNMT3A* mutation (compared with wild type)			
Nonsynonymous variations	1.229 (0.391–3.864)	0.724	NS^*^
Deleterious mutations	2.285 (0.459–11.374)	0.313	NS^*^
R882 mutation	2.285 (0.459–11.374)	0.313	NS^*^
Increasing age	1.023 (0.991–1.055)	0.157	NS
Increrasing BM blasts	1.018 (0.986–1.051)	0.285	NS
SCT performed (compared to not performed)	0.118 (0.035–0.399)	0.001	Good

Note: *P* values were adjusted with *FLT3* ITD mutation status, age, BM blast percentage, and stem cell transplantation performance status (in all 67 patients^*^).

FLT3: fms-related tyrosine kinase 3; ITD: internal tandem duplications; DNMT3A: DNA methyltransferase 3A; R: arginine; BM: bone marrow; SCT: stem cell transplantation; HR: hazard ratio; CI: confidence interval; NS: not significant.
