# Supplementary material for: Incidence and Prognostic Impact of DNMT3A Mutations in Korean Normal Karyotype Acute Myeloid Leukemia Patients
Source: Biomed Res Int. 2015 Jan 11;2015:723682. doi: 10.1155/2015/723682 (PMC4306257; doi:10.1155/2015/723682)

Supplemental figure 1. Schematic 3D structure of DNMT3A protein (A) and detailed site annotation of R736 and R882 residue (B).

Note: Supplemental figure 1A shows schematic 3D structure of DNMT3A protein. The white arrows indicate the sites of R882 (left) and R736 (right) residues. R882 residue resides in the confronting side with another DNMT3A molecule and target DNA. In contrast, R736 residue is located on the helix in the opposite side. Two DNMT3A molecules conform dimer to be functional DNA (Used gene model**:** GRCh37.p5, NT_022184.15, NM_175629.1, NP_783328.1, forward, plus strand). Supplemental figure 1B shows detailed site annotation of DNMT3A mutations (indicated with yellow color). R882 residue is located in the helix near to the 3A-3A interface (indicated with green and purple color) but R736 residue is located toward DNMT3L (indicated with blue and brown color).

Abbreviation: R, arginine.

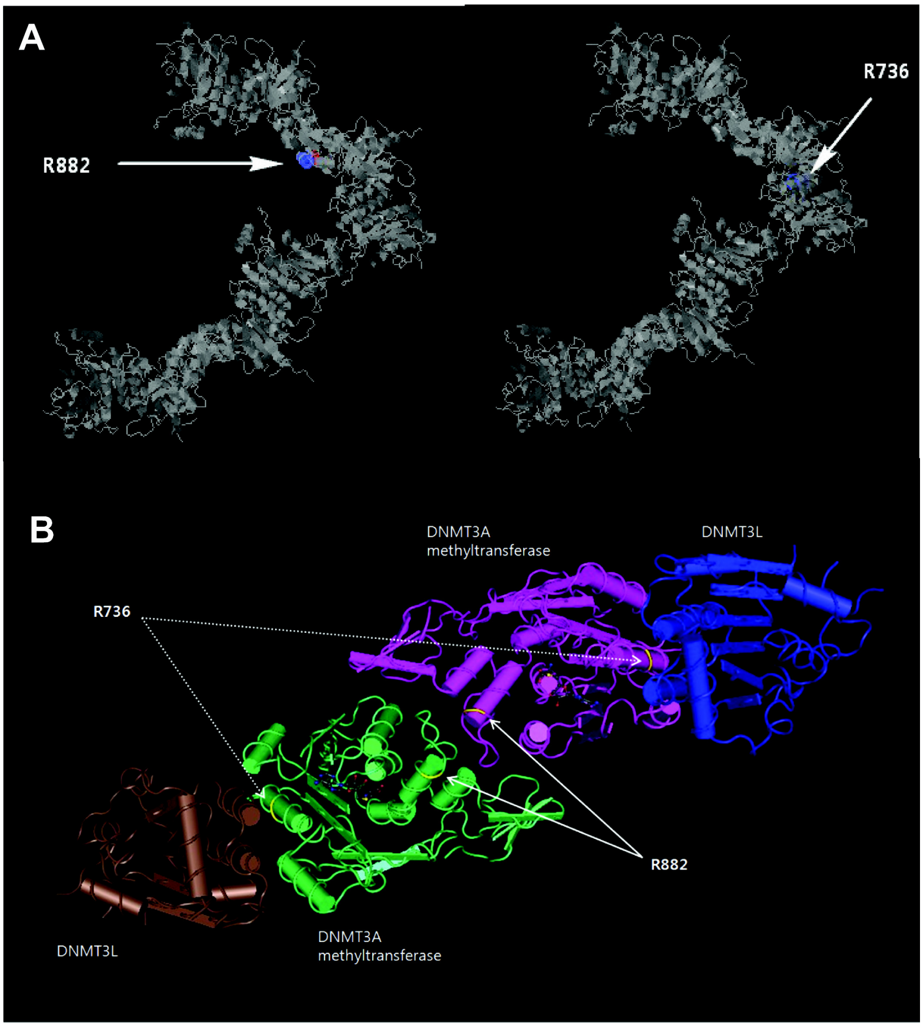

Supplement: Supplementary file 1 — Schematic 3D structure of DNMT3A protein and detailed site annotation of R736 and R882 residue [file 723682.f1.pdf]
